# Jada System Versus Bakri Balloon for Postpartum Hemorrhage: A Retrospective Cohort Study

**DOI:** 10.7759/cureus.115872

**Published:** 2026-09-06

**Authors:** Chukwudera Okolo, Melanie Lagomichos, Courtney Shaver

**Affiliations:** 1 Obstetrics and Gynecology, Baylor Scott & White All Saints Medical Center, Fort Worth, USA; 2 Obstetrics and Gynecology, Anne Burnett Marion School of Medicine at Texas Christian University, Fort Worth, USA; 3 Biostatistics, Baylor Scott &amp; White Research Institute, Dallas, USA

**Keywords:** bakri, intrauterine balloon tamponade, intrauterine tamponade device, jada, postpartum hemorrhage, quantitative blood loss, uterine atony, vacuum-induced hemorrhage control

## Abstract

Introduction: The main objective of this study was to determine whether a single intrauterine tamponade device (Bakri balloon or Jada system) offers superior efficacy and improved clinical outcomes in patients with postpartum hemorrhage. Materials and methods: We conducted a retrospective chart review of 232 unique patient encounters in which postpartum hemorrhage was diagnosed and an intrauterine tamponade device was utilized. We then analyzed particular variables of interest to compare overall clinical outcomes. Results: There were no statistically significant differences in the outcomes of interest between the two intrauterine tamponade devices, suggesting comparable effectiveness of both modalities in clinical practice. Conclusions: Blood loss and blood product transfusion were similar between the two intrauterine tamponade devices. Likewise, there was no statistically significant difference in rates of escalation to surgical intervention and intensive care unit admission.

## Introduction

Postpartum hemorrhage is defined by the American College of Obstetricians and Gynecologists as a cumulative blood loss greater than or equal to 1,000 mL or blood loss accompanied by signs or symptoms of hypovolemia within 24 hours after the birth process [1]. Postpartum hemorrhage is a relatively common cause of maternal morbidity and mortality worldwide, with the rate of postpartum hemorrhage increasing from 2.7% of delivery hospitalizations in 2000 to 4.3% in 2019 [2]. Worldwide, hemorrhage is the leading direct cause of maternal death (accounting for 27.1% of maternal deaths), with the majority of reported hemorrhage deaths classified as postpartum hemorrhage [3]. In the United States, postpartum hemorrhage accounted for 12.1% of maternal deaths between 2017 and 2019, while obstetric hemorrhage was the leading cause of pregnancy-related death (i.e., 25%) in 2019 in the state of Texas [4]. The majority of maternal deaths and severe maternal morbidity attributable to obstetrical hemorrhage are preventable. This highlights the importance of efficiently and effectively identifying and treating postpartum hemorrhage in the clinical setting.

The most common etiologies of postpartum hemorrhage include uterine atony (accounting for 7-80% of cases), retained placental tissue, obstetric trauma, and coagulopathy [1]. Early evaluation and identification of the etiology of postpartum hemorrhage allows for initiation of appropriate treatment and is paramount to decreasing severe maternal morbidity and mortality. Postpartum hemorrhage secondary to uterine atony, identified by palpation of a soft, poorly contracted uterus, can be managed in several ways. Uterotonic agents (e.g., oxytocin, methylergonovine, misoprostol, and 15-methyl prostaglandin F2α) can be used to prompt contraction of the postpartum uterus [5]. If bleeding persists despite utilization of these initial measures, uterine tamponade devices and surgical intervention (e.g., pelvic artery embolization, uterine compression sutures, or hysterectomy) may be considered.

Uterine tamponade devices are used as a second-line therapy in the treatment of atony-related postpartum hemorrhage before escalation to surgical intervention. It is theorized that adequate hemorrhage control is achieved with this method via direct compression of the uterine vessels [6] and/or due to direct mechanical pressure that inhibits bleeding from the spiral arteries [7]. Uterine balloon tamponade devices have been the mainstay of uterine tamponade techniques, with the Bakri balloon being the first and best studied [8]. More recently, uterine vacuum devices have been developed, with the Jada system currently being the only commercially available device in the United States. Both aforementioned intrauterine tamponade devices have undergone individual assessment. Real-world utilization and effectiveness of the Jada system were assessed in the RUBY trial, which concluded that use of the device provided rapid and effective bleeding control, with treatment success rates of 92.5% for vaginal births and 83.7% for cesarean births [9]. A meta-analysis of studies using the Bakri balloon to treat postpartum hemorrhage reported an overall pooled success rate of 85.9% (87% for vaginal deliveries and 81.7% for cesarean deliveries) [10]. However, few data directly compare clinical outcomes in patients treated with the two intrauterine tamponade devices. The purpose of this study was to compare clinical outcomes of patients treated with the Bakri balloon and Jada system for the management of postpartum hemorrhage. This was a comparative observational study and was not designed as an equivalence or non-inferiority study; therefore, the absence of statistically significant differences between groups should not be interpreted as evidence of equivalence or non-inferiority.

## Materials and methods

This retrospective observational study was conducted at Andrews Women’s Hospital at Baylor Scott & White, the only dedicated women’s hospital in Fort Worth, Texas, which was recently designated a Level IV maternal care center. Patients aged 18-65 years who underwent a vaginal or cesarean delivery complicated by postpartum hemorrhage were included in the study if an intrauterine tamponade device was used in their clinical management. The study protocol was reviewed and approved by the Institutional Review Board (025-398) on July 16, 2025. The study qualified for expedited review, and the requirement for informed consent was waived because of its observational design. An interactive data visualization software was used to identify delivery hospitalizations complicated by postpartum hemorrhage between January 1, 2020, and July 31, 2025. These delivery hospitalizations were then further filtered to include only those in which an intrauterine tamponade device was utilized as part of the treatment. Information was then collected through a detailed review of the electronic health records for these patient encounters.

The primary aim of this study was to compare clinical outcomes in patients treated for postpartum hemorrhage with an intrauterine tamponade device (Bakri balloon or Jada system) to determine whether one device is superior in efficacy and clinical outcomes. Primary outcomes included (1) total quantitative blood loss, (2) need for blood transfusion (i.e., packed red blood cells), (3) escalation to surgical intervention (i.e., suction dilation and curettage, uterine compression sutures, or hysterectomy), and (4) transfer to the intensive care unit for a higher level of care. Secondary outcomes included (1) transfusion of other blood products (e.g., fresh frozen plasma, platelets, or cryoprecipitate) and (2) postpartum hospital readmission (within 30 days of initial discharge). The data were organized into two overarching categories: (1) cases in which a Bakri balloon was used and (2) cases in which the Jada system was used.

A chi-square test (or Fisher’s exact test when low cell counts were present) was used to test for associations in bivariate comparisons, and a Wilcoxon rank-sum test was used to test for differences in continuous variables between those who used the Bakri balloon versus the Jada system. All statistical analyses were performed in SAS 9.4 (SAS Institute Inc., Cary, NC, USA). This project was classified as a retrospective cohort study, and no outside or commercial funding sources were provided.

## Results

**Figure 1:**
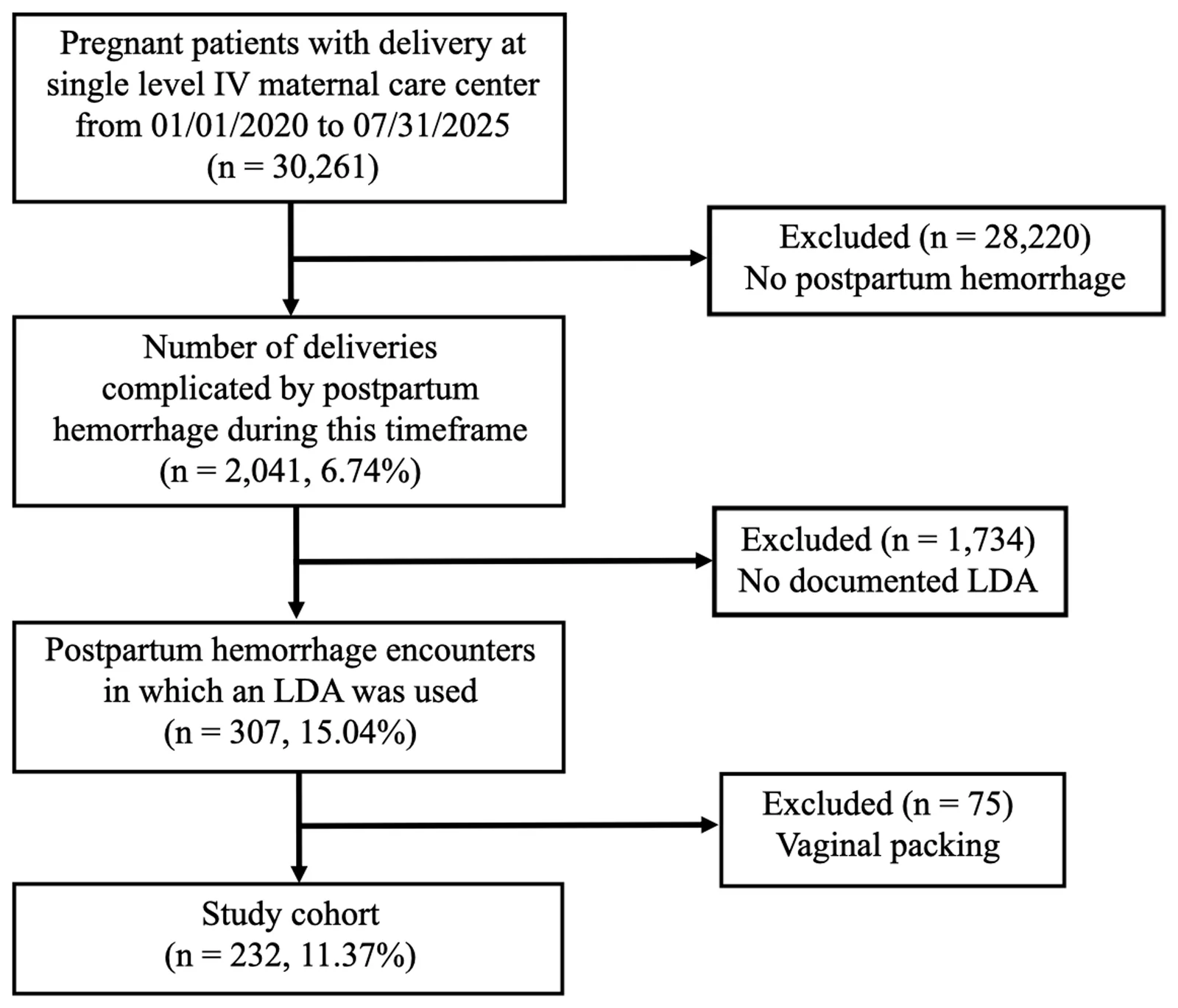
Study population flow diagram

A total of 30,261 deliveries occurred at Andrews Women’s Hospital from January 1, 2020, to July 31, 2025 (Figure 1). Of these deliveries, 2,041 (6.7%) were complicated by postpartum hemorrhage according to ICD-10-CM codes and documented post-delivery quantitative blood loss of 1,000 mL or greater. Of these encounters, 307 had a documented LDA. However, further investigation revealed that intrauterine tamponade devices were used in only 232 (11.4%) postpartum hemorrhage events; the remaining 75 documented LDAs represented vaginal packing. Of the 232 cases, the Bakri balloon was used in 114 (49.14%) encounters, and the Jada system in 118 (50.86%). Baseline demographics, mode of delivery, and antibiotic administration for infection prophylaxis are outlined in Table 1.

**Table 1:** Baseline characteristics for the Bakri balloon and Jada system groups For continuous variables, the median and IQR (p25-p75) were reported, and a Wilcoxon rank-sum test was used for analysis. For categorical variables, the data were presented as n (%) and analyzed using a chi-square test. When cell counts were low for categorical variables, Fisher’s exact test was used and is noted with an asterisk (*). Statistical significance was set at p < 0.05. IQR: interquartile range

Characteristic	Bakri balloon (n = 114)	Jada system (n = 118)	p-value
Maternal age (years)	30 (25-34)	30 (27-34)	0.3683
Gestational age	38.48 (37.29-39.29)	38.57 (37.00-39.43)	0.5284
Race			*0.167
American Indian or Alaska Native	1 (0.88)	1 (0.85)	
Asian	6 (5.26)	3 (2.54)	
Black or African American	13 (11.40)	6 (5.08)	
Missing	7 (6.14)	3 (2.54)	
Other	7 (6.14)	7 (5.93)	
White or Caucasian	80 (70.16)	98 (83.05)	
Mode of delivery			0.5353
Spontaneous vaginal	65 (57.02)	62 (52.54)	
Operative vaginal	1 (0.88)	3 (2.54)	
Cesarean	48 (42.11)	53 (44.92)	
Antibiotic prophylaxis administered			0.2995
Yes	101 (88.60)	99 (83.90)	
No	13 (11.40)	19 (16.10)	

When quantitative blood loss was compared between the two devices, the two groups demonstrated similar total quantitative blood loss (1796 mL (1433-2222 mL) for the Bakri balloon and 1603 mL (1300-2092 mL) for the Jada system, 95% CI (0.976, 1.027), Wilcoxon rank-sum = 2.5089, p = 0.1132). Similar rates of transfusion of packed red blood cells (n = 66, 57.9% vs. n = 64, 54.2%, 95% CI (0.704, 1.983), χ² = 0.3148, p = 0.5747), escalation to surgical intervention (n = 24, 21.1% vs. n = 19, 16.1%, 95% CI (0.714, 2.705), χ² = 0.9413, p = 0.3319), and transfer to the intensive care unit for higher level of care (n = 3, 2.6% vs. n = 6, 5.1%, 95% CI (0.123, 2.068), Fisher’s exact test = 0.1728, p = 0.4997) were also observed (Table 2). When considering the secondary aims of the study, we found similar rates of transfusion of other blood products, including cryoprecipitate (n = 5, 4.4% vs. n = 8, 6.8%, χ² = 0.6281, p = 0.4281), fresh frozen plasma (n = 12, 10.5% vs. n = 16, 13.6%, χ² = 0.5026, p = 0.4783), and platelets (n = 6, 5.3% vs. n = 9, 7.6%, χ² = 0.5358, p = 0.4642). Furthermore, rates of postpartum readmission within 30 days of initial hospital discharge for hemorrhage-related causes showed no statistically significant difference between devices (n = 17, 14.9% vs. n = 19, 16.1%, χ² = 0.0626, p = 0.8025) (Table 3).

**Table 2:** Primary outcomes for all cases in which an intrauterine tamponade device was utilized For continuous variables, the median and IQR (p25-p75) were reported, and a Wilcoxon rank-sum test was used for analysis. For categorical variables, the data were presented as n (%) and analyzed using a chi-square test. When cell counts were low for categorical variables, Fisher’s exact test was used and is noted with an asterisk (*). Statistical significance was set at p < 0.05. QBL: quantitative blood loss, pRBC: packed red blood cell, IQR: interquartile range

	Bakri balloon (n = 114)	Jada system (n = 118)	p-value	Test statistic
Total QBL (mL)	1796 (1433-2222)	1603 (1300-2092)	0.1132	W = 2.5089
Any pRBC transfusion			0.5747	χ² = 0.3148
Yes	66 (57.89)	64 (54.24)		
No	48 (42.11)	54 (45.76)		
Surgical intervention utilized			0.3319	χ² = 0.9413
Yes	24 (21.05)	19 (16.10)		
No	90 (78.95)	99 (83.90)		
Transfer to higher level of care			*0.4997	FET = 0.1728
Yes	3 (2.63)	6 (5.08)		
No	111 (97.37)	112 (94.92)		

**Table 3:** Secondary outcomes for all cases in which an intrauterine tamponade device was utilized For categorical variables, the data were presented as n (%) and analyzed using a chi-square test. Statistical significance was set at p < 0.05.

	Bakri balloon (n = 114)	Jada system (n = 118)	p-value	Test statistic
Transfusion of other blood products				
Cryoprecipitate			0.4281	χ² = 0.6281
Yes	5 (4.39)	8 (6.78)		
No	109 (95.61)	110 (93.22)		
Fresh frozen plasma			0.4783	χ² = 0.5026
Yes	12 (10.53)	16 (13.56)		
No	102 (89.47)	102 (86.44)		
Platelets			0.4642	χ² = 0.5358
Yes	6 (5.26)	9 (7.63)		
No	108 (94.74)	109 (92.37)		
Postpartum readmit			0.8025	χ²= 0.0626
Yes	17 (14.91)	19 (16.10)		
No	97 (85.09)	99 (83.90)		

## Discussion

This retrospective single-center observational study comparing clinical outcomes between patients treated for postpartum hemorrhage with either a Bakri balloon or the Jada system reveals similar results for both devices. The results indicate no statistically significant differences in total quantitative blood loss, need for blood transfusion (i.e., packed red blood cells), escalation to surgical intervention, or transfer to the intensive care unit for a higher level of care. Our findings extend the previous literature comparing the effectiveness of the two devices for hemorrhage control, which was limited to a single study with 666 patient encounters [11]. Furthermore, the outcomes noted in this study are generally consistent with prior publications demonstrating the efficacy of both intrauterine tamponade devices [9,10].

Given the absence of demonstrated superiority of either device in this and prior studies, the decision to use one versus the other may be reasonably influenced by device cost [8,11]. At this institution, the acquisition cost of a single Jada system is $1,295, compared with approximately $301 for a Bakri balloon, a 4.3-fold difference in device cost. Given the absence of measurable clinical benefit and the substantial difference in acquisition costs, routine use of the Jada system may increase healthcare expenditures without a corresponding improvement in patient outcomes. It is worth noting, however, that a shorter indwelling time for the Jada system (median 6 hours) than for the Bakri balloon (median 18 hours) [11] has been reported, which is consistent with manufacturer recommendations for each device. However, the overall length of hospital stay was the same for patients treated with each device. Though this study did not perform a formal cost-effectiveness analysis, these findings highlight the importance of incorporating value-based considerations into device selection and support further investigation into the cost-effectiveness of emerging technologies for postpartum hemorrhage management.

This study has several limitations. First, it was not a randomized controlled trial, and the populations comprising the two device groups may have differed. Additionally, the lack of a standardized postpartum hemorrhage protocol or algorithm limits interpretation of the results. At our institution, more than 50 obstetric delivery providers independently determine care escalation and device selection. Thus, variation in provider practice patterns may have influenced both treatment allocation and clinical outcomes.

It is important to note that at our institution, both the Bakri balloon and Jada system are primarily utilized for the management of postpartum hemorrhage secondary to uterine atony refractory to uterotonic medications rather than hemorrhage caused by obstetric trauma, retained placental tissue, or coagulopathy. Consequently, we anticipated that the etiology of postpartum hemorrhage was uterine atony for the overwhelming majority of patients in whom either device was used. For this reason, etiology-specific analyses were not performed in this study. Nevertheless, because hemorrhage etiology was not independently verified or abstracted, we cannot rule out the possibility that a subset of patients had mixed or alternative etiologies, which represents another limitation of this study. Additionally, this study did not adjust for hemorrhage severity at the time of device placement, including blood loss prior to intervention, timing of device deployment, or markers of hemodynamic instability. Consequently, differences in baseline clinical severity may have influenced outcomes independent of device selection.

**Figure 2:**
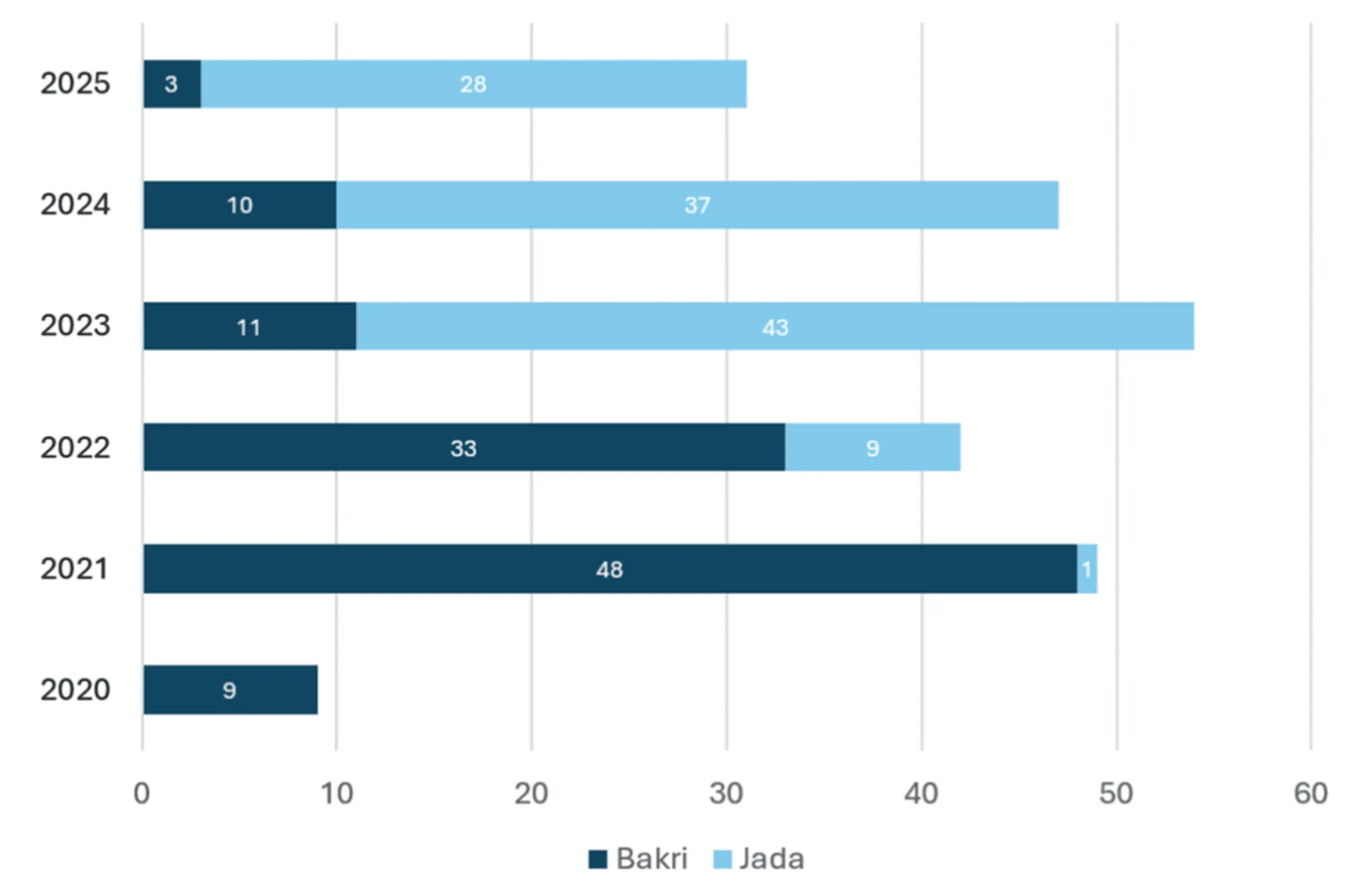
Device utilization stacked bar graph

Furthermore, device utilization was not uniform throughout the study period; in fact, yearly utilization data show a clear transition from preferential use of the Bakri balloon to the Jada system. The Bakri balloon was used primarily during the early years of the study, whereas the Jada system became predominant in the later years (Figure 2). As a result, another limitation of this study is the susceptibility to temporal bias. Changes in postpartum hemorrhage management practices, provider familiarity with hemorrhage treatment options, and operator experience over time may have influenced clinical outcomes independent of device selection. Since the two devices were not used concurrently to the same extent throughout the study period, it is difficult to determine whether the observed outcomes are attributable to the individual devices themselves or to broader changes in clinical practice and patient care.

## Conclusions

We found that both the Bakri balloon and the Jada system performed similarly when used in the treatment of postpartum hemorrhage. Our data suggest no statistically significant difference in clinical outcomes between the two intrauterine tamponade devices. In the absence of observed or proven superiority and given the substantial cost difference between the devices, these findings raise important considerations regarding value-based resource utilization in obstetric care. Nevertheless, randomized controlled trials utilizing a standardized postpartum hemorrhage algorithm and standardized measures of hemorrhage severity are needed to better assess the comparative effectiveness and cost-effectiveness of these devices.
