# Correction: Jada System Versus Bakri Balloon for Postpartum Hemorrhage: A Retrospective Cohort Study

**DOI:** 10.7759/cureus.c480

**Published:** 2026-09-17

**Authors:** Chukwudera Okolo, Melanie Lagomichos, Courtney Shaver

**Affiliations:** 1 Obstetrics and Gynecology, Baylor Scott & White All Saints Medical Center, Fort Worth, USA; 2 Obstetrics and Gynecology, Anne Burnett Marion School of Medicine at Texas Christian University, Fort Worth, USA; 3 Biostatistics, Baylor Scott &amp; White Research Institute, Dallas, USA

This article has been corrected to state the following: "At this institution, the acquisition cost of a single Jada system is $1,295, compared with approximately $301 for a Bakri balloon, a 4.3-fold difference in device cost." The originally reported cost reflected the price of three Jada devices rather than a single unit.

